# Dr. John M. Turner’s Anomalous Cases of Pregnancy, with Remarks

**Published:** 1842-12-03

**Authors:** John M. Turner

**Affiliations:** Barnwell District, (S. C.)


### *Anomalous cases of Pregnancy; with Remarks.*

By John M. Turner, M. D.

As we have thought nothing more calculated to stimulate the minds of medical practitioners, to a farther and deeper research in the profession, than the relation of rare and uncommon cases, (accompanied with remarks,) we have concluded to give a brief sketch or history of the two following, which have fallen under our observation within the present year, and which at the time somewhat excited our curiosity.

*Case 1st.*—Mrs. H———, a female of small stature, about thirty years of age, of delicate constitution, and mother of several children, became pregnant; her pregnancy, however, was borne with more difficulty, or as she expressed it, with more suffering than any of her former pregnancies, so that she looked forward to the time of her accouchement, with some degree of evil foreboding.

Her labour at length came on, but was delayed a month or longer, beyond the time of calculation; was conducted with tardiness and considerabte irregularity in the uterine efforts. After passing through a somewhat tedious labour, however, and without sustaining any permanent injury, a living fœtus was expelled, but which was immediately followed (by the expulsive efforts of the same pain) by a large *fleshy mass* about four inches in length, and nearly the size of a man's arm. In due length of time, about thirty minutes, the placenta and membranes were safely detached, and no untoward symptom afterwards, to mother or child.

The only thing uncommon in the above case, was the expulsion of the fleshy mass which did not bear any particular marks of organization or life, though, when cut into, appeared to be partly like muscle half macerated in water, and another part almost like a diseased tumour of a scirrhous nature. One portion of it seemed as if it adhered pretty firmly to the interior of the uterus; the other part was covered with a thin skin, somewhat granular in appearance, more like the skin covering the breast of a turkey or chicken, than any thing else I can compare it to. It was not attached to the placenta in any way that I could discover. Not having an opportunity to weigh it, I cannot give the exact weight, but suppose it was nearly two pounds.

*Case 2d*, was similar in many respects to the one just described. It was a coloured woman about thirty years of age, (property of a gentleman in my neighbourhood.) She had borne several children, and from the unusual size of the abdomen, was thought to be pregnant with twins. Her gestation was borne with difficulty; and much alarm, combined with evil apprehensions concerning her approaching confinement, haunted her mind continually.

The time of her accouchement at length arrived, and as in case first, the labour was slow and tedious, from which circumstance, and its being a presentation of the feet, I was sent for in haste. When I arrived, the midwife had brought down the feet, legs, andbody of the fœtus, in which condition I found it, with the arms and cranium lying pretty firmly locked in the maternal pelvis. I learned it had been in this situation more than half an hour; without much difficulty I extricated the arm that lay imbedded in the hollow of the sacrum, and the expulsion of the cranium soon followed by the next uterine effort; but as I had expected and predicted on my arrival, from finding the umbilical cord cold and circulation ceased or arrested, from the pressure it had so long sustained, the life of the fœtus was extinct.

As soon almost as the fœtus was delivered with the next pain that followed, and before the placenta, came a large *mass* surrounded with coagulated blood, which on being examined bore pretty much the appearance of the one described in case first. Instead of being the same length, it was more rounded and a little larger than the first, having the appearance on one side as if it adhered to the interior of the uterine cavity by a kind of pedicle, and covered nearly all over with a skin of the granulated appearance as the other; but when cut into with a scalpel, instead of putting on so much the appearance of a *fleshy fibrous* mass, looked more like a diseased substance, being tough, tuberculated, and semi-cartilaginous throughout.

This being the second case of the kind I have met with in one year, and having come across no other like it in eight years previous practice, I was resolved to investigate more particularly the nature of the substance, and consequently availed myself of the opportunity of procuring it; which I did, and now have it preserved in alcohol, a fine specimen for a museum. It weighs a small fraction over two pounds, and seems to be studded through some part of it with little concretions, almost like tubercles. It is curious in both cases; the mass or substance was totally unconnected with the placenta or fœtus, but seems from the appearance of one side of it, to have adhered firmly to the cavity of the uterus. That cases do occur occasionally in which the uterus contains and expels substances of a fleshy, bloody, fibrinous or aqueous nature, while in a diseased condition, or as a consequence of a blighted conception, we have ample evidence; but for such to occur at the end of a healthy gestation, and that too when we find the fœtus perfect, as far as my observation extends, we think uncommon and rare; and in fact as far as our reading extends, we do not recollect exactly a similar case. The nearest to the point I have recently seen in print, is related by Dr. Meigs of Philadelphia, which is about as follows. (Vide Medical Examiner, page 185, 1st volume, March 19th.) After relating a case of natural labour, he says—"Attached to the edge of the placenta was another mass of an indurated structure, like a gland; to this gland-like substance was a pedicle or chord which enlarged, and which contained at its extremity a mass or substance shrivelled, and looking like a mummy." Dr. Meigs thinks his was a case of twins, in which one of the twins had perished at about two months after conception, while the other survived throughout the full period of utero-gestation. Another case of a nature similar to the one referred to by Dr. Meigs, will be found recorded in the "Lancet," for September, 1836, and extracted from it by the Editor of the Southern Medical and Surgical Journal, where Mr. Owen Fox relates a case which fell under his observation and care, of a female that had been recently delivered of a fine healthy child, when after waiting a short time, and during a strong pain, he made slight extension on the cord, while the placenta, together with the mass, came away. On examining the latter, he found it to consist of a *small fœtus* about five inches in length, which he considered to be a parturition of a child at full time, and a blighted fœtus. The question then suggests itself—Would the same opinions as entertained by Dr. Meigs and Mr. Fox, in these cases, be applicable to the cases I have above related? Though the cases of these gentlemen approach nearer to such as I have given, than any I have seen in print, still the difference is too great, we think, to arrive at exactly a similar inference; for it is evident that the substance or mass mentioned by these gentlemen, had all the evidence of organization, or that they had been truly vitalized bodies, possessing a regular form and shape, which I could not detect in the two cases I have given.

From the great dissimilarity then existing in the true nature of the substances expelled, which we find does exist when we come to examine them nicely, we have thought that ours were genuine cases of conception of one fœtus that was carried to full maturity, while the mass that was expelled directly after the full grown fœtus, was what is termed a *false conception* or *mole;* that is the consequence of a real conception, which took place at the time or soon after the genuine impregnation, but which perished directly or before the period that nature begins to fashion or form it into any regular shape; but which, notwithstanding the loss of vitality, continued to grow and become converted into that peculiar fleshy fibrous mass already described. Professor Rigby says—"Where any cause has occurred to destroy the life of the embryo during the *early weeks* of pregnancy, one of two results follows, either that expulsion takes place sooner or later, or the membranes of the ovum become remarkably *changed*, and continue to grow for some time longer, until at length they form a fleshy fibrous mass, called mole, or false conception. Again, La Motte says—" Every mole is a blighted ovum, which has been the product of conception." Further—It is well known that the vinous absorbing radicles of the chorion, which give it that shaggy appearance during the first months of pregnancy, are the means by which the embryo is furnished with a due supply of nourishment at this period. If the embryo should die from any cause, and the uterus show no disposition to expel the ovum, the nourishment which has been collected by the absorbing power of the chorion, appears now to be directed to the chorion itself, which therefore puts on a fleshy and sometimes fibrous growth, and increases rapidly in size.

Such we think to be the true physiological explanation of the formation of mole or false conception. And if the mass that we have described be the consequence of a blighted ovum, where did the conception of the same take place? At the time of the genuine conception that carried a fœtus to maturity, or after, while pregnancy was progressing? Here arises the question of superfœtation or double conception, of which we have many cases recorded by Professor Dunglison in his physiology, and consequently we have thought that possibly it took place after the genuine conception, and if so, was probably the main cause of its premature decay and change of structure, which we have described, on the principle that the most vitalized portion of the maternal blood being sent to the support of the first conception, the latter must naturally decay, or at least lose its vital principle. It might perhaps be suggested by some that the substance we have described partook of the nature of tubercle or some such diseases or tumours that affect the uterine cavity. But then we would ask the question, how could an individual labouring under disease go through a healthy utero-gestation, and bring to full purfection a healthy and living fœtus, when the nature of such diseases are to wear down the strongest constitutions? That it was something connected with pregnancy and utero gestation we think admits of no doubt, as the subjects in both cases gave no evidence of any uterine affection, existing previous to conception, nor since delivery. If it was truly mole existing with true pregnancy, it seems something novel in obstetric medicine.

*Barnwell District*, (S. C.) Nov., 1842.

